# Time tracking and multidimensional influencing factors analysis on female breast cancer mortality: Evidence from urban and rural China between 1994 to 2019

**DOI:** 10.3389/fpubh.2022.1000892

**Published:** 2022-09-26

**Authors:** Xiaodan Bai, Xiyu Zhang, Wenjing Xiang, Yanjie Wang, Yu Cao, Guihong Geng, Bing Wu, Yongqiang Lai, Ye Li, Baoguo Shi

**Affiliations:** ^1^Department of Economics, School of Economics, Minzu University of China, Beijing, China; ^2^Research Center of Health Policy and Management, School of Health Management, Harbin Medical University, Harbin, China

**Keywords:** breast cancer, urban areas, rural areas, Age-Period-Cohort model, prediction, the theory of social determinants of health

## Abstract

**Background:**

There are huge differences in female breast cancer mortality between urban and rural China. In order to better prevent breast cancer equally in urban and rural areas, it is critical to trace the root causes of past inequities and predict how future differences will change. Moreover, carcinogenic factors from micro-individual to macro-environment also need to be analyzed in detail. However, there is no systematic research covering these two aspects in the current literature.

**Methods:**

Breast cancer mortality data in urban and rural China from 1994 to 2019 are collected, which from China Health Statistical Yearbook. The Age-Period-Cohort model is used to examine the effects of different age groups, periods, and birth cohorts on breast cancer mortality. Nordpred project is used to predict breast cancer mortality from 2020 to 2039.

**Results:**

The age effect gradually increases and changes from negative to positive at the age of 40–44. The period effect fluctuates very little and shows the largest difference between urban and rural areas in 2019. The birth cohort effect gradually decreases with urban-rural effects alternating between strong and weak. In the predicted results, the urban-rural mortality gap becomes first narrow and then wide and shows a trend of younger death.

**Conclusions:**

From the perspective of a temporal system, the changing trend of breast cancer mortality is highly consistent with the history of social and economic structural changes in China. From the perspective of the theory of social determinants of health, individuals, families, institutions and governments need to participate in the prevention of breast cancer.

## Introduction

Breast cancer (BC) always ranks in the top 10 among Chinese woman's cancer deaths, and its ranking is still rising at the beginning of the new century (1). According to the International Agency for Research on Cancer (IARC) of the World Health Organization (WHO), the morbidity of BC (19.9%) ranks first of cancer among Chinese women, and the mortality rate (9.9%) ranks fourth among all cancers in 2020 (2), women live in fear of BC. On the one hand, BC has many causative factors, including congenital heredity (3–6), acquired habits or lifestyle (4, 7–9), and living environment (4, 10–12), crises surrounding everywhere. On the other hand, after experiencing the multiple shocks of economic, psychological and medical conditions (4), the probability of diagnosed patients surviving has been reduced. This anxiety is exacerbated by the huge disparity between China's urban and rural areas (13).

The urban-rural dual system is a major feature of China's development. Rural areas are dominated by agriculture, while urban areas are more inclined toward higher-paying service industries. As a result, the development of the two regions is not always balanced. The display in Table 1 proves this. Based on the statistics of differences in population and facility quality, the values of some health indicators (health workers, number of beds in medical institutions, and maternal and child health hospitals) are higher in rural areas than in urban areas. But the overall narrowing trend of the development gap of health indicators between urban and rural areas. Also, the growth rate of the gap in the per capita disposable income of residents has decreased gradually, especially after the rural revitalization strategy was proposed in 2017.

**Table 1:** Development differences between urban and rural China in 2010–2019.

	**Population (10,000 people)**		**Per capita disposable income of** **residents (Chinese Yuan** ¥**)**		**Health workers (1,000 people)**		**Number of beds in medical institutions (1,000)**		**Maternal and child health hospitals (one)**		**Infant mortality (1/1,000)**	
	**Urban Areas**	**Rural Areas**	**Urban Areas**	**Rural Areas**	**Urban Areas**	**Rural Areas**	**Urban Areas**	**Rural Areas**	**Urban Areas**	**Rural Areas**	**Urban Areas**	**Rural Areas**
2010	66,978	67,113	19,109.4	5,919.0	3,647.861	4,549.641	2,302.297	2,484.534	1,042	1,983	5.8	16.1
2011	69,079	65,656	21,809.8	6,977.3	3,844.201	4,761.839	2,475.222	2,684.667	1,042	1,994	5.8	14.7
2012	71,182	64,222	24,564.7	7,916.6	4,141.058	4,967.647	2,733.403	2,991.372	1,058	1,986	5.2	12.4
2013	73,111	62,961	26,467.0	9,429.6	4,488.500	5,291.983	2,948.465	3,233.426	1,096	2,048	5.2	11.3
2014	74,916	61,866	28,843.9	10,488.9	4,770.661	5,453.652	3,169.880	3,431.334	1,096	2,002	4.8	10.7
2015	77,116	60,346	31,194.8	11,421.7	5,127.704	5,556.177	3,418.194	3,597.020	1,120	1,958	4.7	9.6
2016	79,298	58,973	33,616.2	12,363.4	5,487.317	5,675.628	3,654.956	3,755.497	1,145	1,918	4.2	9.0
2017	81,347	57,661	36,396.2	13,432.4	5,892.116	5,846.856	3,922.024	4,018.228	1,160	1,917	4.1	7.9
2018	83,137	56,401	39,250.8	14,617.0	6,263.898	6,026.427	4,141.427	4,262.661	1,166	1,914	3.6	7.3
2019	84,843	55,162	42,358.8	16,020.7	6,665.163	6,253.172	4,351.540	4,455.416	1,168	1,903	3.4	6.6

Data from China Statistical Yearbook and China Health Statistical Yearbook.

Additionally, the China Health Statistical Yearbook announced that the mortality rates of BC for urban and rural women in China were 9.34/100,000 and 6.92/100,000 in 2019 (1), an increase of 14.69 and 47.55% respectively compared to the end of the 1990's. Rural women worry that local medical resources are limited and that cancer is found in the late stage without effective treatment (14). Urban women worry that their immunity would continue to decline due to the strange vision and huge psychological pressure that come with the diagnosis (15). To reduce the mortality rates of BC, it is necessary not only to find causative factors for “prevention” but also to focus on the fairness of resources for “treatment,” so that more women can escape the shadow of BC.

Research on carcinogenic factors has been very rich so far. Global burden of disease (GBD) research divides risk factors into four dimensions, mainly focusing on the extraction of population characteristics, namely environmental and occupational, behavioral, metabolic and dietary risks (16–18). However, based on the theory of social determinants of health, the influencing factors of disease occurrence cover more dimensions. If a person eats too much high-fat diets, the steroids in it will be converted into estrogen in the body, which promotes the formation of BC cells (19); Increasing spending power is a sign of wealthier people, and disease prevention is more effective among themselves (20); Agriculture has been the backbone of China's economy for a long time in history, women often worked on the farmland, and more exercise could change the way estrogen is metabolized and reduce the risk of BC (21); Aromatic hydrocarbon receptors mediate the effects of many endocrine disruptors in polluted environments and have implications for BC in young or premenopausal women (22), and so on.

Coincidentally, Age-Period-Cohort (APC) analysis may be used to better depict the entire complex of social, historical, and environmental factors that simultaneously impact individuals or social groups (23). Therefore, it is widely used to assess the characteristics and quality of the epidemic trend of various diseases. At present, there are three major breakthroughs in BC related literature using the APC model: the expansion of regional scope (24), the long traceability of time (21), and the focus on causative factors (25). Wang et al. distinguished between Chinese urban and rural areas and compared with South Korea, Japan, and the United States, finally they summarized the similarities on pursuing health and the differences on cultural habits in different ages and periods of eastern and western society. Ding et al. used the net effect of the birth cohort in the APC model to measure the risk of cancer death for the time where no death data were collected (1906–1990), it verified that its trend was consistent with major political and socioeconomic events in China since the 20th century. Based on the fact that the impact of educational inequality on the mortality rate of BC in Korea changed from positive to negative, Bahk et al. conducted APC analysis by focusing on educational level. The result demonstrated that an increasing trend in BC mortality among Korean women between 1983 and 2012 was due to the increased mortality of the lower education groups, not the highest education group. This also shows that the application of the APC model has a huge space for meticulous processing.

Nevertheless, there are still some deficiencies in the previous literature. First, the comparative analysis of BC deaths focusing on urban and rural areas is not rich enough, but China has a vast territory and a large population, and the problem of urban-rural dual structure is prominent (26). Failure to make a comprehensive and precise distinction may exacerbate the inequity between urban and rural areas (27). Second, the time horizon is concentrated on the past, but rarely on the future. The cohort effect in the APC model is useful for understanding the development of relevant BC over a long period of time in the past, ignoring predict its change in the future that we cannot see. However, the predicting trend has an important guiding role. For example, Yasmeen F predicted a continued increase in the morbidity of BC in U.S. for black and white women over the next 15 years, and this increase would be faster in black women, thus making recommendations to improve high-quality care for black people (28). Katayama and Narimatsu predicted that BC cases in the urban area of Kanagawa, Japan, among people over 65 years old would be peak by 2040 (31.2% increase from 2010), such a high increase in demand for BC treatment naturally required a lot of medical resources (29). Third, the attribution dimension of BC deaths in urban and rural areas is limited. The four dimensions of risk factors summarized by GBD ignored the role of the macro environment. Moreover, most studies explained the phenomenon that the deaths in urban areas increased more slowly than in rural areas from the perspective of better access to health care (24). There are also studies on the goal of reducing mortality rates by curbing high morbidity. They point out that urban lifestyles such as high-fat diets, less exercise, and later childbirth tend to be more westernized, so urban women have higher morbidity rates (19). However, these studies lack emphasis on a more comprehensive perspective from other factors. This causes people to ignore other equally important lethal factors and reduce their chances of surviving.

In such a context, our study has some outstanding innovations. On the one hand, considering history as a mirror, we apply the APC model to trace the historical trend of BC mortality rate changes in urban and rural China to avoid the recurrence of high levels of the risk factors in the past. At the same time, we also pay attention to the future, Nordpred project is used to predict the future, which can obtain more precise results by correlating mortality with age structure and population scale (30). That is to say, we characterize BC deaths in a long time context from the past to the future. To our knowledge, no study has been published covering such a long temporal system. Moreover, in order to make up for the lack of comprehensive discussion on non-pathological factors such as economic and social factors in previous studies, the analysis of BC causative or lethal factors in urban and rural areas are widely covered. Supported by the theory of social determinants of health, comprehensive determinants of BC are covered from personal biological characteristics to group living environment, social environment and social development perspectives. It is expected to provide a theoretical reference for intervening in the future trend of continued increase in BC mortality, and also to provide suggestions for resource allocation under the mortality turning trend, which is of great significance for providing BC the prevention evidence for countries with urban-rural differences like China.

## Materials and methods

### Data sources and processing

Following the data requirements of the APC model, this paper collects data on BC mortality in the age group of 20–84 years old women in urban and rural China from 1994 to 2019. The mortality rate (1/100,000) is calculated as the proportion of BC deaths in the total population, and these data are obtained from China Health Statistical Yearbook (1995–2020). Moreover, these data are processed as follows: (1) About age: due to the rare occurrence of cancer in the population before the age of 20, and the patients over the age of 85 have low immunity and usually die from other complex causes. What's more, the APC model cannot handle the open age group (31), only rates for those within the age range of 20–84 are considered here, yielding 13 5- year age groups. (2) About period: 5-year average data commonly used in previous APC modeling studies (32, 33), the dilemma is that people in the 5-year-old age group in 5- year period end up being born over a 10-year period, and continuing to estimate the birth cohort effect this way reduces the temporal resolution by a half (34). Therefore, this paper uses single-year data with an interval of 5 years. Specifically, we analyze age-group mortality data for 1994, 1999, 2004, 2009, 2014, and 2019 to ensure that women in the 5-year-old age group within 1 year were all born within the 5 years in the past. (3) About birth cohort: according to different age groups in the specific study period, we can trace the earliest date of birth to 1910–1914 (year 1994 – age 84 = birth year 1910; and year 1994 – age 80 = birth year 1914); and the latest to 1995–1999 (year 2019 – age 24 = birth year 1995; and year 2019– age 20 = birth year 1999), yielding 18 birth period groups. Further, the “unidentifiable” problem in the APC model should be solved, so this paper uses the intrinsic estimator (IE) algorithm (31) to identify the three variables of age, period, and cohort, which can be used to estimate linear and nonlinear components.

There are stringent requirements for data processing by using the Nordpred project to predict future female BC mortality rates. First, the project predicts the mean BC mortality rate for women in different age groups in the unknown period group based on the mean number of BC deaths by the existing period group. We choose 1996–2019 from the existing 1994–2019 data to ensure evenly grouping, yielding 6 4-year groups, and we plan to predict 2020–2039, a total of 5 groups of data. Second, BC mortality is calculated based on the female population structure. So we collect the population forecast data of 18 age groups of urban and rural women from 1996 to 2039 and divide them into a group every 4 years to take the average. Population forecast data comes from the Department of Economic and Social Affairs of the United Nations (35). Finally, population age standardization is performed. The data comes from the WHO World Standard (36) publicity. All data above processing is performed in Excel 2010 software.

### Age-Period-Cohort model

Traditional methodologies are often unable to overcome the problem of interaction between time-related influencing factors when describing the epidemiological characteristics of diseases. The APC model just makes up for this shortcoming. It controls to some extent the interaction among the effect of age, period, and birth cohort on trends in disease mortality (37), making the trend of cancer clearer. The mathematical theory of the APC model is based on the Poisson distribution (38), and the basic expression of the Poisson log-linear model is as follows:


(1) $$ln(M_{ijk})=ln\left(\frac{D_{ijk}}{P_{ijk}}\right)=\text{μ}+{\text{α}}_{i}+{\text{β}}_{j}+{\text{γ}}_{k}+{\text{ε}}_{ijk}$$

Where *M*_*ijk*_ denotes the mortality rate of female BC in the age group i, the time period j, and the birth period k, which is calculated as the ratio of the number of deaths from BC in women (*D*_*ijk*_) to the total number of women exposed to BC risk (*P*_*ijk*_). *μ* denotes the intercept; *α*_*i*_ denotes the coefficient of age group i (i = 20-24, ……, 80-84); *β*_*j*_ denotes the coefficient of time period j (j = 1994, ……, 2019); *γ*_*k*_ denotes the coefficient of birth cohort k (k = 1910-1914, ……, 1995-1999); *ε*_*ijk*_ denotes random sampling error and obeys standard normal distribution.

Equation (1) can be viewed as a multiple regression model, where the three regression coefficients in the model are the net effect of each regression coefficient after controlling for the other two regression coefficients. Equation (1) is subject to only one constraint (21):


(2) $$\begin{array}{l} \sum {\text{α}}_{i}=\sum {\text{β}}_{j}=\sum {\text{γ}}_{k}=0 \end{array}$$

APC analysis is performed by using the apc_ie package in STATA (version 15.0).

### Nordpred project

Nordpred project can use the Poisson Age-Period-Cohort (APC) models to calculate prediction of BC mortality. The predictions are relatively reliable due to its correlation with age structure and population scale. The prediction process can refer to the description of the data processing section, and it is carried out in the open-source software R (version4.1.1).

## Results

### Dynamic statistics of breast cancer mortality in urban and rural women in China from 1994 to 2019

**Figure 1:**
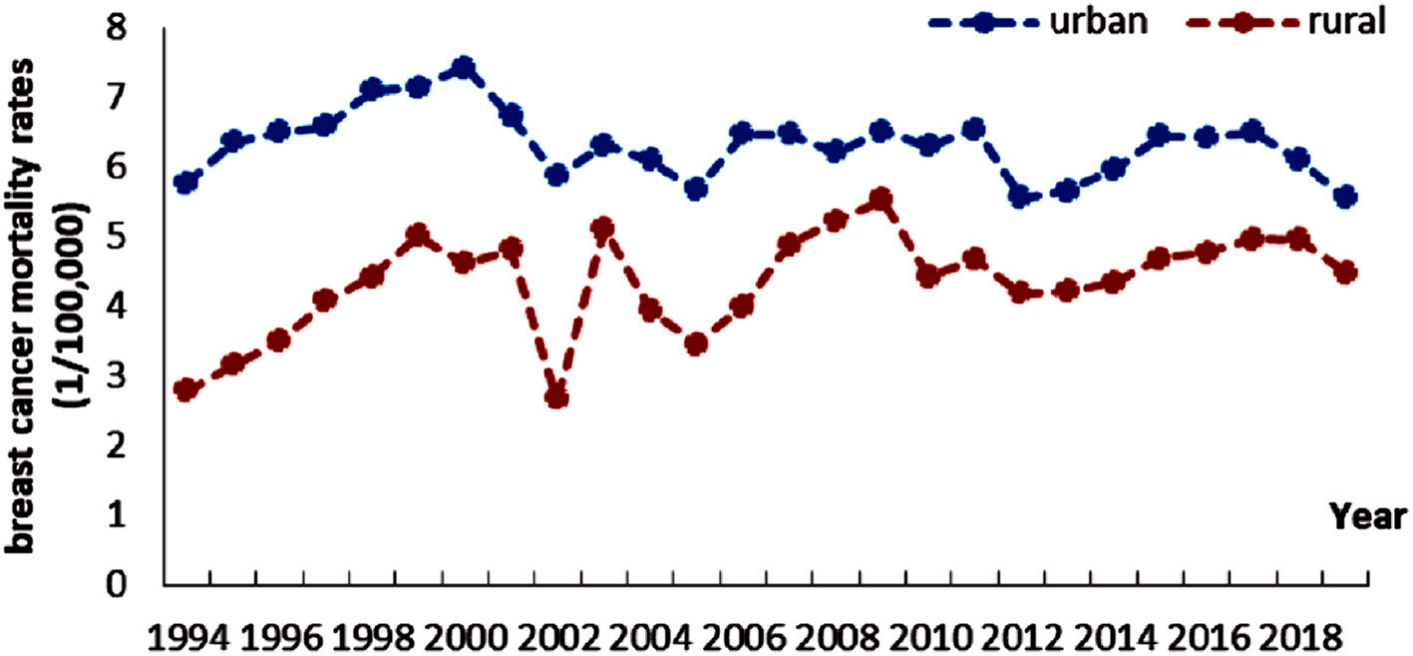
Breast cancer mortality rate for women aged 20–84 years old, 1994–2019, urban and rural China.

Figure 1 shows the annual mortality of BC in urban and rural women in China from 1994 to 2019. In general, the change trends of the two areas are similar. The mortality rates in 2002, 2005, 2010, and 2012 all formed a “v”-shaped structure with their respective adjacent years. However, there are obvious differences, the urban mortality rate is always higher than that of the rural areas, but the range of change in urban areas is lower than that of the rural areas. It is worth noting that although these mortality rates reflect time trends over the past 30 years, the depicted trends may be biased by women's chronological age and birth year.

**Figure 2:**
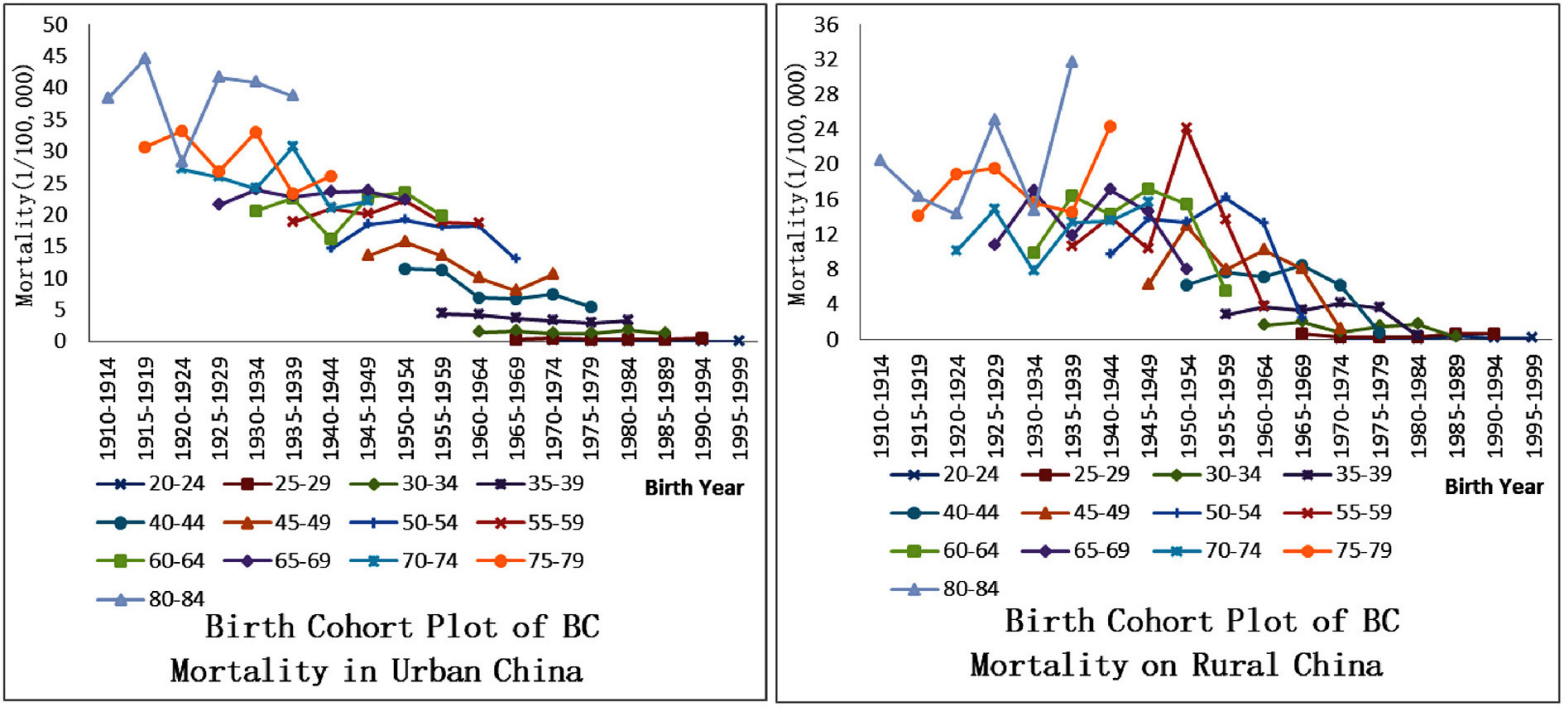
Birth cohort plot of breast cancer mortality among urban and rural women, China.

Figure 2 can just prove this point, that is, there is a significant difference in BC mortality rates from the perspective of women's age and birth year, indicating the existence of effect from age and birth year on the risk of BC death in Chinese women. In urban areas, the mortality rate for women aged 80–84 who were born in 1910–1914 is the highest (except in 1920–1924), and the rates are the lowest for women aged 20–24 who were born in 1995–1999. In short, it generally follows the pattern of “when the birth period is earlier, the mortality rate will increase with the increase of age.” But this pattern is not obvious in rural areas. The BC mortality rate of women over 40 years old in rural areas fluctuates greatly under the change of birth year. The APC model can extract age and birth cohort effects from the overall temporal trend, allowing a more precise assessment of temporal trends in BC mortality.

### Age-Period-Cohort modeling results in urban and rural areas

**Figure 3:**
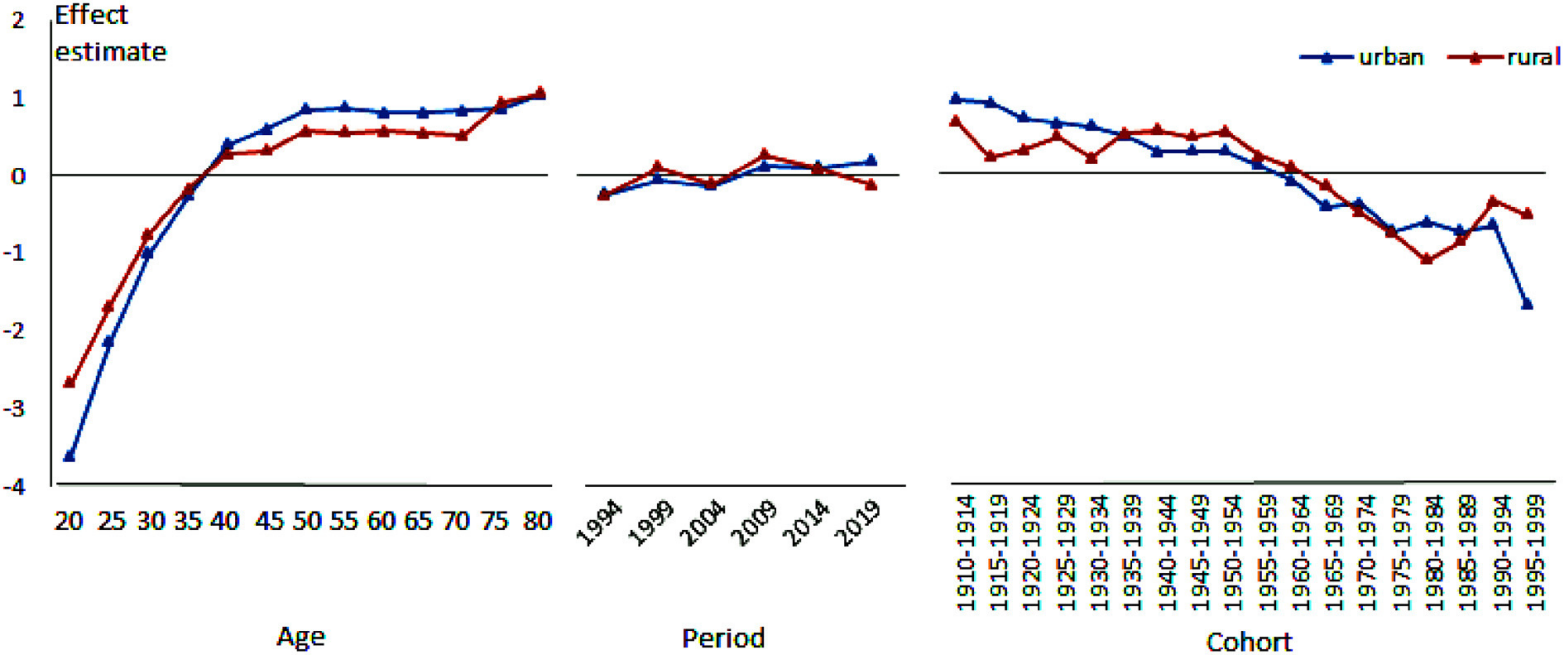
Age, period and cohort effect on breast cancer mortality in urban and rural China.

Table 2 summarizes age, period, and cohort effect estimates for urban and rural China, respectively. The estimated effects provide a measure of adjusted risks of BC mortality by age/year. We plot the contents of the table as a graph and get Figure 3.

**Table 2:** APC model analysis results of BC mortality rates for women in urban and rural China.

**Age**	**Urban**	**Rural**	**Period**	**Urban**	**Rural**	**Cohort**	**Urban**	**Rural**
20–24	−3.622 (1.281)	−2.682 (0.964)	1994	−0.239 (0.116)	−0.251 (0.129)	1910–1914	0.949 (0.338)	0.666 (0.300)
25–29	−2.152 (0.594)	−1.693 (0.553)	1999	−0.050 (0.085)	0.116 (0.097)	1915–1919	0.911 (0.303)	0.213 (0.247)
30–34	−1.013 (0.390)	−0.761 (0.379)	2004	−0.133 (0.071)	−0.104 (0.090)	1920–1924	0.716 (0.288)	0.308 (0.213)
35–39	−0.268 (0.304)	−0.177 (0.300)	2009	0.120 (0.068)	0.265 (0.081)	1925–1929	0.640 (0.278)	0.476 (0.183)
40–44	0.399 (0.247)	0.272 (0.243)	2014	0.111 (0.088)	0.092 (0.100)	1930–1934	0.606 (0.275)	0.200 (0.180)
45–49	0.598 (0.212)	0.318 (0.212)	2019	0.191 (0.111)	−0.118 (0.124)	1935–1939	0.483 (0.278)	0.519 (0.169)
50–54	0.846 (0.178)	0.562 (0.177)				1940–1944	0.279 (0.292)	0.559 (0.185)
55–59	0.868 (0.151)	0.554 (0.154)				1945–1949	0.296 (0.307)	0.471 (0.209)
60–64	0.800 (0.133)	0.563 (0.138)				1950–1954	0.296 (0.325)	0.540 (0.229)
65–69	0.808 (0.122)	0.544 (0.133)				1955–1959	0.112 (0.349)	0.221 (0.265)
70–74	0.828 (0.123)	0.517 (0.140)				1960–1964	−0.090 (0.376)	0.078 (0.295)
75–79	0.863 (0.135)	0.933 (0.144)				1965–1969	−0.415 (0.411)	−0.155 (0.333)
80–84	1.045 (0.157)	1.050 (0.170)				1970–1974	−0.377 (0.442)	−0.500 (0.389)
						1975–1979	−0.730 (0.507)	−0.762 (0.468)
						1980–1984	−0.618 (0.581)	−1.098 (0.644)
						1985–1989	−0.728 (0.767)	−0.863 (0.787)
						1990–1994	−0.651 (0.216)	−0.345 (1.025)
						1995–1999	−1.679 (4.854)	−0.528 (2.460)
cons	1.891 (0.293)	1.555 (0.165)						
AIC	4.8649	4.9453						
BIC	−184.034	−146.032						

The values in this table are the regression coefficients of the three effects, and the standard errors are in parentheses.

#### The age effect of breast cancer mortality

The age effect of BC mortality in both urban and rural women in China shows an inverted “J” shape with age, which indicates that the risk of BC death in women gradually increases with age, and it also shows that the rate of change in mortality with age is slow in the old, comparing the faster rate of change in the young. What's more, starting from the age of 40, the age effect of BC mortality in both urban and rural women increases to a positive value, which means that for women over 40 years old, age is a risk factor to BC mortality. In addition, there are also differences in the age effect of BC mortality between urban and rural areas. In the age group below 40 years old (negative effect stage), the absolute value of the urban age effect coefficient is always larger than that in rural areas (showing that the urban curve is below the rural curve), and also in the age group above 40 years old (positive effect stage) as does (showing that the urban curve is above the rural curve). On the one hand, it shows that the age effect of BC mortality in urban women increases significantly faster than in rural areas. On the other hand, it also shows that when age becomes a risk factor of BC mortality, urban women are at higher risk.

#### The period effect of breast cancer mortality

The period effect of BC mortality in Chinese urban and rural women generally shows an “M” shape with time, that is, it shows two repeated fluctuations of “up-down.” Moreover, the period effect difference in BC mortality among urban and rural women is relatively tiny. The most significant difference appears in 2019. The urban period effect is larger than that of the previous period (2014), while the rural areas keep a continuous downward trend or even drop to a negative value on the basis of the previous period (2014).

#### The birth cohort effect of breast cancer mortality

The birth cohort effect of BC mortality in both urban and rural areas in China generally shows a downward trend with birth year, and change from positive value to negative value during 1960–1969, that is, for women who were born before this period, their birth year is a risk factor to BC mortality. In addition, when the birth year acts as a risk factor, the cohort effect in urban areas changes from strong to weak, and the cohort effect in rural areas is just the opposite; When birth year is the protective factor, the cohort effect in urban areas changes more rapidly than in rural areas, showing that the inhibitory effect is stronger in urban areas for most of the periods.

### Prediction of breast cancer mortality in urban and rural women in China from 2020 to 2039

**Figure 4:**
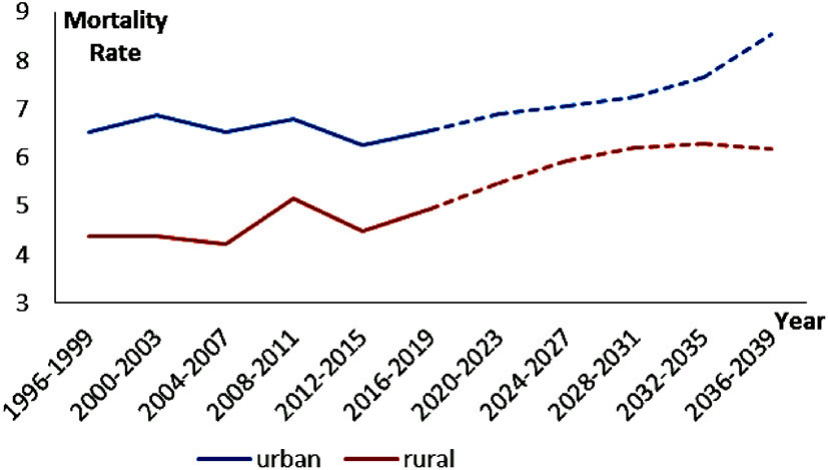
Prediction of breast cancer mortality in Chinese women (age standardization).

Figure 4 shows the age-standardized BC mortality rates prediction results for Chinese urban and rural women by using the Nordpred project. Whether in the past or the future, the BC mortality rate for urban women has always been higher than that in rural areas, and future projections for both areas are on upward trends. Over the forecast period of the next 20 years, the gap in BC mortality between the two areas shows a trend of gradually narrowing and then rapidly widening. (1) 2020–2031: the gap is gradually narrowing. During this period, the rising rate of urban mortality has slowed significantly, while the rising rate of rural mortality has just accelerated. (2) 2032–2039: the gap is rapidly widening. At this time, the rate of mortality growth in urban areas accelerates significantly, but in rural areas, it becomes slow and even has a slow downward trend after reaching the highest value in 2032–2035.

**Figure 5:**
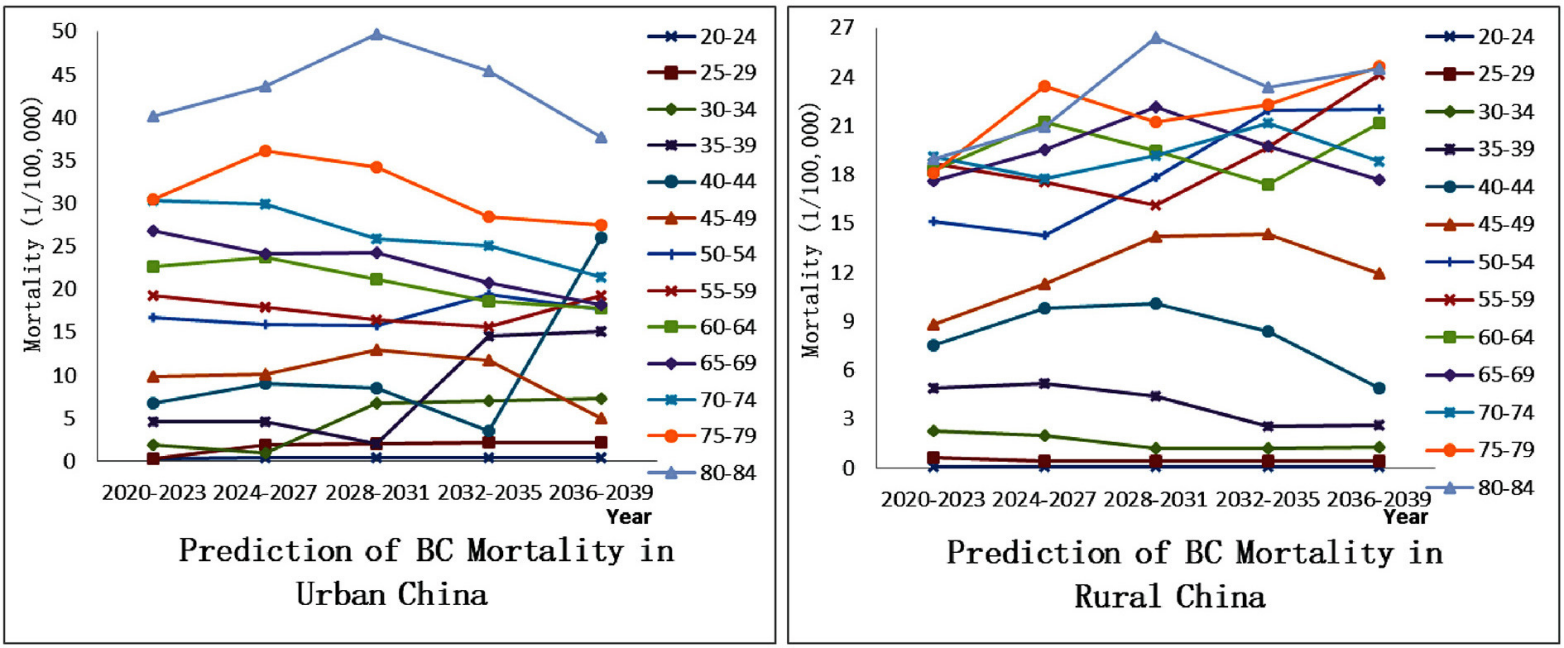
Prediction of breast cancer mortality in Chinese women (different age groups).

To more accurately judge the difference between the overall trend and the trend of female BC mortality in different age groups, we also plot the predictions of BC mortality in Chinese urban and rural women by age group, as shown in Figure 5. From the perspective of urban areas, there is a pattern that “the mortality rate of BC increases with age.” However, it is obvious that relatively young women (25–29, 30–34, 35–39, 40–44, etc.), rather than older women (the trend is just the opposite), are in line with the trend in total mortality from 2020 to 2039 (Figure 4). From the perspective of rural areas, the female mortality rate in the 20–54 age group shows the same pattern as the urban one, but the situation in the 55+ age group does not conform to this pattern. Moreover, similarly, the BC mortality rate of younger women (25–29, 30–34, 35–39, 40–44, 45–49,etc.) and the total mortality rate are closer to the trend of change from 2020 to 2039, indicating that the BC mortality rate in the future is more inclined to young people.

## Discussion

This paper collects BC mortality data for women aged 20–84 years in urban and rural China from 1994 to 2019 and uses the APC model to extract the net effects of age, period, and birth cohort. At the same time, the Nordpred project is used to predict the trend of BC mortality in urban and rural areas in the next 20 years. Moreover, a multi-dimensional analysis is made for the exploration of BC determinants. Our study has great significance. First, under the background of China's urban-rural dual structure, the unequal allocation of various resources and the difference in the change of BC mortality between the two areas are typical representations. At the same time, China is continuously narrowing the urban-rural gap through programs such as “rural revitalization” and “common prosperity,” which can provide a reference for other BC high-incidence regions in the world with uneven development. Second, through the APC model and the prediction results, we construct a long temporal system that includes the past, present and future for the first time, taking history as a guide to continuously improve health and allow more and more women to escape the shadow of BC. This is something that previous research has not covered. Third, under the framework of the theory of social determinants of health, we divide a clear structural level from the micro-individual to the macro-environment, making up for the lack of theoretical framework of previous research, and reflecting the importance of joint efforts by different subjects such as individuals, communities, and governments to change the current situation of high morbidity and high mortality of BC.

### The complete temporal system: Past, present and future

The birth cohort effect extracted by the APC model can be traced back to a relatively early period, and it can show the changing trend of BC mortality during 1910–1999 under the data frame of this paper. We find that the birth cohort effect weakened with birth period and it shows the characteristics of alternating strong and weak in urban and rural areas. This is actually a reflection of the changes in ideology, the inheritance of traditional culture, the reform of the medical system and the development of health technology that accompany the transformation of China's social and economic structure. The founding of the Republic of China in 1912 opened a new chapter of women's liberation (39). The rapid development of agriculture before 1949 gradually improved women's diets, especially in rural areas where “natural advantages” (21), In the early days of the founding of the People's Republic of China, the government-led medical security system began to play a role (40), and the cooperative medical system, which was rapidly promoted nationwide in the 1960's (41), became one of the driving forces for the transformation of the positive and negative attributes of the birth cohort effect. Since the reform and opening up in 1978, the medical system reform has been on the right track, and the medical technology has been also becoming more advanced, urban areas with relatively mature and complete facilities gained the upper hand at this time. However, the loss of the “collective economy” made it more difficult for the rural areas where the development of health organizations has been slow (42). Simultaneously, the development of science and technology in China has made rapid progress. The combination of it and the medical field (43) has also ushered in the dawn of BC screening and treatment. All these changes have found a breakthrough in the prevention or treatment of BC. However, the elderly who were born at a relatively early period happened to be the ones who experienced this series of “earth-shaking” changes, the traditional ideas and concepts have been deeply ingrained, and their ability and willingness to accept new things are relatively low, so these changes actually have a very limited impact on the elderly.

The period effect extracted by the APC model can be viewed as the present change in BC mortality. Overall, the period effect of BC mortality in urban and rural areas has only a small fluctuation. This largely depends on which of the BC protective factors and risk factors prevail over time. BC mortality has a downward trend when protective factors such as improvements in medical technology and people's lives play leading roles. However, BC mortality has an upward trend when risk factors such as environmental pollution, bad living habits, and job competition pressure play leading roles. Partially, the biggest difference between urban and rural period effects occurred in 2019. The rural revitalization plan (44) in recent years has made great contributions to narrow the gap between urban and rural areas, which has improved the situation in all aspects of the countryside and achieved remarkable results in curbing the high mortality rate of diseases.

**Figure 6:**
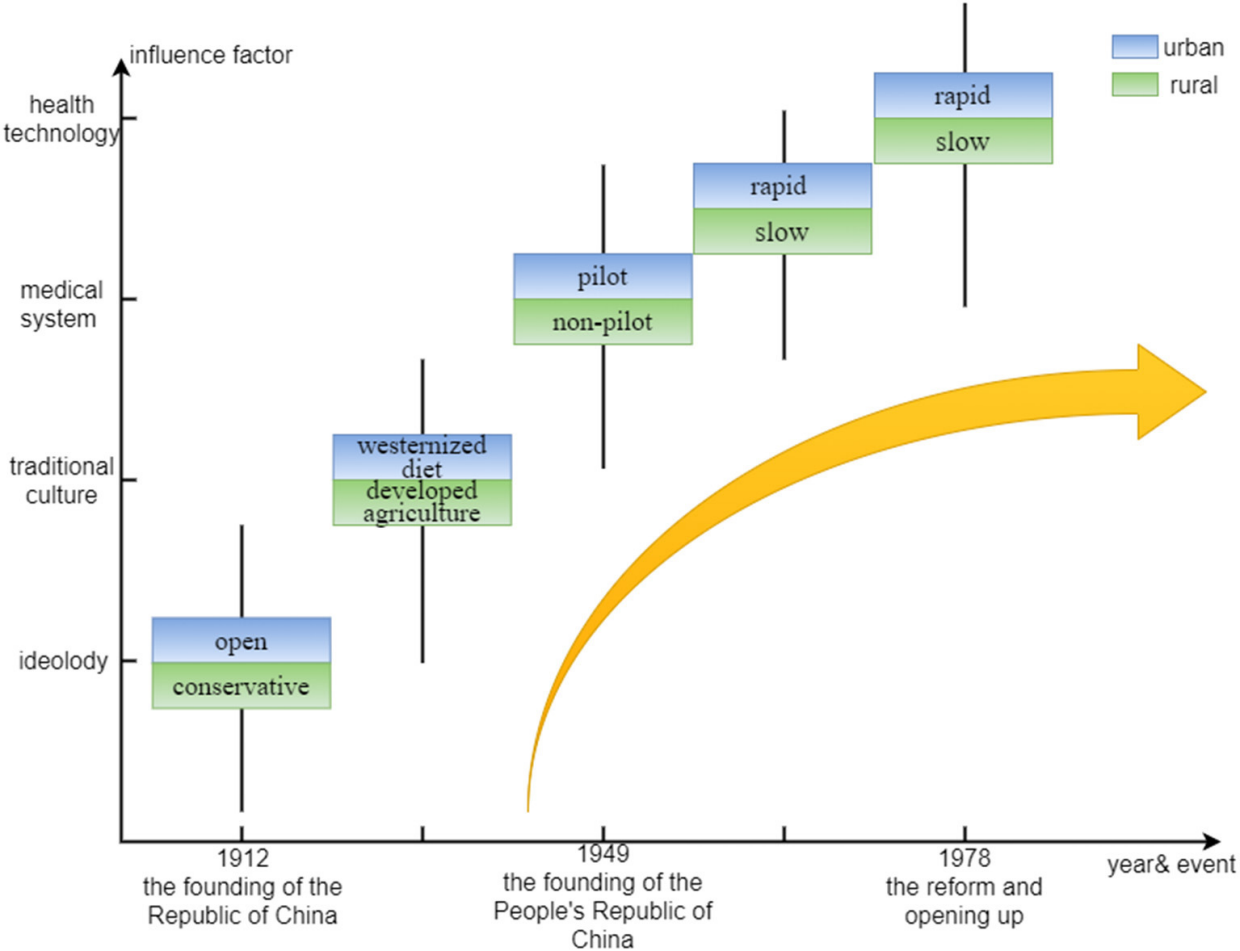
Influencing factors of breast cancer in urban and rural areas in the course of social development in China.

Prediction of BC mortality over the next 20 years has great significance. (1) The stage in which the urban-rural mortality gap is gradually narrowing. This may be the result of the accumulation of rural risk factors over time. Since the 1980's, farmers have chosen to work in cities to increase their income. On the one hand, due to their low level of education and lack of special skills, they had to engage in high-risk jobs such as construction and textile industry; On the other hand, these farmers are not official residents of cities, and most of them are excluded from the city's medical and social security benefits. Coupled with the restriction of the household registration policy, if there are BC patients among these people, they should be registered in the rural areas rather than the city where they live (13). (2) The stage in which the urban-rural mortality gap is rapidly widening. With the reform of the household registration system (45), a large number of farmers can flock to cities and even settle down to enjoy the city's medical benefits. The population base of cities becomes larger, and the morbidity and mortality of BC will increase. In addition, the role of some “big city diseases” should not be ignored. (3) BC mortality rates in both urban and rural areas will be more skewed toward younger groups in the future. This may be related to the higher morbidity caused by the more westernized lifestyle of young people in the future (19), and the extremely high morbidity causes extremely high mortality. Figure 6 shows influencing factors of BC in urban and rural areas in the course of social development in China.

In such a long temporal system, we deeply understand that although the worsening result of BC ultimately manifests as the death of an individual, it is the product of different environmental backgrounds constructed at various stages of social development. The societal change affects the activities of micro-individuals, making BC different between individuals, and individuals in turn affect their living environment in order to adapt or change this state. To discuss the influence of micro-individual and macro-environment more clearly, we rely on the theory of social determinants of health to analyze various carcinogenic factors according to the level.

### The theory of social determinants of health: From the micro-individual to the macro-environment

The theory of social determinants of health divides the influencing factors of disease into the six parts.

(1) Age and genetic factors. The age effect extracted by the APC model is one of the most basic features in the analysis of population heterogeneity, and it is also the innermost ring factor in the hierarchical model of health social influencing factors established by Dahlgren and Whitehead in 1991. Our results show that BC mortality increases with age in urban and rural China, which is consistent with the accumulation of time exposure to risk factors and the decline of bodily functions (especially immunity) in the elderly. At the same time, the age group of 40–44 is the key point that age changes from a protective factor to a risk factor. On the one hand, women in this age group are under the dual pressure of family and work. On the other hand, they are on the verge of menopause, when the human body is relatively sensitive to changes in hormones (46), making it easier to induce BC. In addition, Monticciolo et al. did research on genetics-based increased BC risk and calculated the lifetime risk of 20% or more (5).

Additionally, from the perspective of histological and molecular classification of BC, there are obvious age-clustering characteristics of special types of BC. According to Renan et al. medullary carcinoma usually affects patients aged 30–40 years, while women aged 50–60 years are more affected by cribriform carcinoma and tubular carcinoma. Mucinous carcinoma, neuroendocrine carcinoma, and invasive lobular carcinoma are more common in the elderly. Also, postmenopausal women are more affected by metaplastic carcinoma and apocrine carcinoma (47). Based on comprehensive gene expression profile studies, the four molecular subtypes of BC include Luminal A, Luminal B, enriched HER2 (HER2+), and Triple Negative (48–50). At the same time, they also show differences in age structure. Anna et al. found that women aged 20–39 years more often had HER2+ and Triple Negative tumors, while women aged 70–89 years more often had luminal A-like tumors (51). Different histological and molecular classifications of BC have different prognosis and treatment response, and the mortality rate is also quite different. Therefore, every link in BC diagnosis, prognosis, drug target, and treatment response prediction is crucial.

(2) Personal lifestyle. Many obvious bad behaviors in urban areas can greatly increase BC risk, resulting in higher BC mortality rates in urban areas than in rural areas. For example, the average age of marriage postponed from 23.4 in the 1990's to 27.1 in the first decade of the 21st century, the breastfeeding rate fell from 67% in 1998 to 28% in 2014 (21), increased beef and pork intake and decreased vegetable intake (52), and the more serious psychological stress and depression (53), etc. In addition, there is evidence of quantitative analysis, including “a 10 g increase in daily alcohol consumption is associated with a 7.1% increase in BC risk” (54), and “the sedentary behavior is significantly positively associated with BC risk” (55). The late marriage age, reduced breastfeeding (56), high fat intake (57), huge psychological pressure, high drinking rate, and lack of physical activity are all more obvious manifestations of urban areas than rural areas with relatively conservative ideas.

(3) Social support network. With the development of the economy, China's health service supply has also achieved a double leap in quality and quantity, and the popularization of medical insurance has provided a guarantee for the prevention and treatment of BC. However, it cannot be denied that the development of medical and health technology needs a long time, and the medical support currently in the period of progress cannot exert its maximum function. As a priority area for the promotion of various medical and health policies, urban areas have advantages over rural areas. In addition, the publicity of BC-related knowledge is insufficient, and people's access to scientific information is very limited, especially in rural areas. Liu et al. conducted a questionnaire on BC self-screening awareness and found that women's awareness of BC is very low (58), which would increase women's unconscious exposure to more carcinogenic factors.

(4) Socioeconomic status. In general, urban women have higher levels of income and education. When people's income is higher, their prevention and treatment of diseases have a money basis, and higher education levels can make them more likely to accept the relevant prevention knowledge and disease treatment process (13).

(5) The nature and environment of work. Research has shown that when young women work long-term night shifts, their BC risk increases by 2.15 times (12). Working at night can cause a disturbance in the biological clock and abnormal estrogen levels in the body. Moreover, changes in light at night may affect the normal circulation of melatonin (8), which increases the BC risk. In addition, some specific occupations have a higher incidence of BC, such as medical staff, teachers, company employees, etc. (59, 60). These people are often stressed and have many night shifts.

(6) Macro-social and economic development and environmental status. In many cases, individual efforts alone cannot escape the risk factors in the macro-environment. In urban areas, rapid economic development can be projected onto advances in the medical field, such as the upgrading of medical equipment, the improvement of medical technology, and the cultivation of medical talents, which has an advantage over rural areas in slowing or halting the upward trend in BC mortality. However, the environmental pollution problems brought about by urban development have weakened its advantages. For example, PM2.5 contains endocrine-disrupting pollutants, which can potentially affect breast density by interfering with the growth of breast cells and increasing the relative number of fibrous tissue (61), promoting the occurrence of diseases.

**Figure 7:**
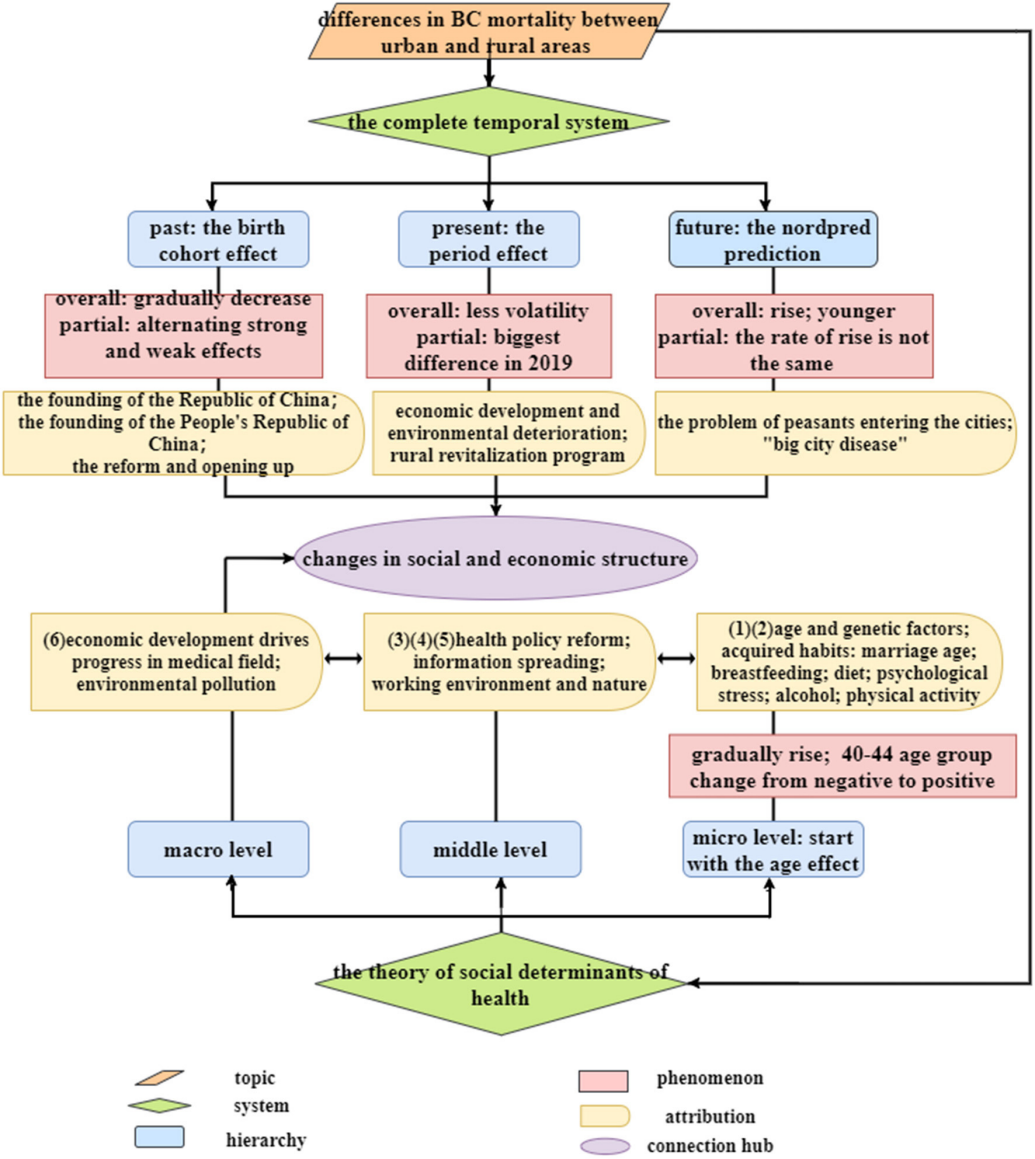
The construction process of the temporal system and the theory of social determinants of health.

In conclusion, the theory of social determinants of health profoundly reveals that the occurrence of BC is affected by multi-level factors, and high-level factors will play a role by affecting low-level factors. It also points out that reducing BC risk requires a concerted effort by many parties. Figure 7 shows the construction process of the temporal system and the theory of social determinants of health.

### Limitation

This paper also has some limitations. First, limitations of the APC model approach: the estimates of the two birth cohort effects are unreliable. For example, the mortality rate for women aged 80–84 in 1994 is the only data source for estimating the cohort effect for 1910–1914; the mortality rate for women aged 20–24 in 2019 is the only data source for estimating the cohort effect for 1995–1999. Second, limitations of data availability: this paper selects annual data. Although there is a time span of nearly 30 years, there are only two types of study areas (urban and rural areas). It is unavoidable that the quantitative analysis bias is too large due to the small sample size. Therefore, we only conduct qualitative analysis. Despite its limitations, this paper provides useful ideas and policy recommendations for BC prevention and treatment.

## Conclusion

Using the APC model and the Nordpred project, our study first collates the BC mortality changes in urban and rural Chinese women in a long temporal system. BC risk is the result of mapping changes in China's socioeconomic structure to individuals and is also the result of catalysis or mitigation of individual different activities. Therefore, with the basis of the theoretical framework of social determinants of health, we have clarified the difference between the innate genetics and acquired habits of micro-individuals, the role of communities, institutions, and families at the middle level, and the impacts of macro-environment. The inequality between urban and rural areas in these aspects has become an important reason for the difference in BC risk. To this end, we make the following recommendations.

First, the inequality between urban and rural areas needs to be addressed urgently. Most of the urban high mortality is due to its high morbidity, so urban areas should focus on BC prevention. The rapidly increasing mortality rate in rural areas is more caused by inadequate screening in the early stage, lack of understanding of relevant information, and untimely treatment in the later stage, so rural areas should focus on BC screening and treatment. In addition, with the advancement of programs such as “rural revitalization” and “common prosperity,” the resolution of urban-rural inequality in China is just around the corner. Advances in health care can help prevent and treat BC, despite the increased risk factors that come with development. Therefore, giving all women equal access to high technology for prevention and treatment would be an effective way to break the positive relationship between development and BC risk (62). Second, attention should be paid to the issue including mortality rates rising and skewing toward younger people in the forecast. Appropriate inclination of medical resources to rural areas is a direct means to intervene in the rapid increase in the mortality rate of rural BC in the next 10 years. At the same time, in order to avoid the sudden increase of urban BC mortality due to the accumulation of early risk factors, solving the problem of farmers entering the city and “big city disease” should be put on the agenda. Last, reducing BC risk requires a concerted effort. Everyone should try their best to maintain a healthy lifestyle and a positive attitude; Families and communities should create a harmonious and warm atmosphere, provide more emotional support and do a good job of publicity about BC prevention; Governments should make overall plans in terms of education level improvement, special occupational attention, medical welfare security, and sustainable economic and environmental development. Only in this way can the goal of human health be achieved.

## Data availability statement

The original contributions presented in the study are included in the article/supplementary material, further inquiries can be directed to the corresponding authors.

## Author contributions

XB is responsible for framework design and writing-original draft. XZ is responsible for data analysis. WX is responsible for visualization. YW, YC, GG, BW, and YLai are responsible for data collection and literature retrieval. BS and YLi are responsible for supervision and writing—review and editing. All authors contributed to the article and approved the submitted version.

## Funding

This work was supported by Humanities and Social Sciences Foundation of Ministry of Education of China (Grant No. 19YJCGAT004), National Social Science Foundation of China (Grant No. 20BGJ026), the project of “Culture, Port Culture and the Land Port Areas in Consolidating the Sense of Community for the Chinese Nation” by Minzu University of China (MUC) (Grant No. 2021MDZL15), and the National Natural Science Foundation of China (Grant No. 71874045, 71403073, and 72174047).

## Conflict of interest

The authors declare that the research was conducted in the absence of any commercial or financial relationships that could be construed as a potential conflict of interest.

## Publisher's note

All claims expressed in this article are solely those of the authors and do not necessarily represent those of their affiliated organizations, or those of the publisher, the editors and the reviewers. Any product that may be evaluated in this article, or claim that may be made by its manufacturer, is not guaranteed or endorsed by the publisher.

## Abbreviations

BC: Breast Cancer
IARC: the International Agency for Research on Cancer
GBD: Global Burden of Disease
APC: Age-Period-Cohort
IE: intrinsic estimator
WHO: World Health Organization.
